# Situs ambiguus in a Brown Swiss cow with polysplenia: case report

**DOI:** 10.1186/1746-6148-9-34

**Published:** 2013-02-20

**Authors:** Alois Boos, Hans Geyer, Urs Müller, Jeanne Peter, Tanja Schmid, Christian Gerspach, Matteo Previtali, Maja Rütten, Titus Sydler, Colin C Schwarzwald, Elisabeth M Schraner, Ueli Braun

**Affiliations:** 1 Institute of Veterinary Anatomy, Vetsuisse Faculty, University of Zurich, Winterthurerstrasse 260, 8057, Zurich, Switzerland; 2 Department of Farm Animals, Vetsuisse Faculty, University of Zurich, Winterthurerstrasse 260, 8057, Zurich, Switzerland; 3 Institute of Veterinary Pathology, Vetsuisse Faculty, University of Zurich, Winterthurerstrasse 268, 8057, Zurich, Switzerland; 4 Equine Department, Vetsuisse Faculty, University of Zurich, Winterthurerstrasse 260, 8057, Zurich, Switzerland

## Abstract

**Background:**

Laterality defects are rare in cattle and usually manifest as asplenia or polysplenia syndrome. These syndromes may be associated with situs ambiguus, which is a dislocation of some but not all internal organs. The objective of this report was to describe the clinical and post-mortem findings including the macroscopic and microscopic anatomy of selected organs in a cow with polysplenia and situs ambiguus.

**Case presentation:**

A 3.5-year-old Brown Swiss cow was referred to the Department of Farm Animals, Vetsuisse Faculty, University of Zurich, because of poor appetite and recurrent indigestion. A diagnosis of situs ambiguus was based on the results of physical examination, ultrasonography, exploratory laparotomy and post-mortem examination. The latter revealed that the rumen was on the right side and lacked compartmentalisation. There were two spleens, one on the left (26.5 x 12.0 cm) and one on the right (20.5 x 5.5 cm), and the omasum was located craniolateral to the ruminoreticulum on the left. The abomasum was located on the right, although it had initially been displaced to the left. The three-lobed liver occupied the left and central cranioventral aspect of the abdominal cavity (cavum abdominis). Only the right and left hepatic veins (vena hepatica dextra and sinistra) drained into the thoracic segment of the caudal vena cava (vena cava caudalis), and histological changes in the liver were indicative of impaired haemodynamics. The mesojejunum was not fused with the mesentery of the spiral loop (ansa spiralis) of the ascending colon (colon ascendens). The latter was folded and the transverse colon (colon transversum) ran caudal to the cranial mesenteric artery (arteria mesenteria cranialis). Fibrotic constrictions were seen in the lumen of the caecum and proximal loop (ansa proximalis) of the ascending colon. Both kidneys were positioned retroperitoneally in a lumbar position. The lumbar segment of the caudal vena cava did not descend to the liver and instead drained into the right azygous vein (vena azygos dextra).

**Conclusions:**

Recurrent digestive problems and poor production in this patient may have been caused by a lack of rumen compartmentalisation, abnormal abomasal motility, constrictions in the large intestine (intestinum crassum) and fibrosis of the liver. The abomasum had abnormal motility most likely because it was anchored inadequately and only at its cranial aspect to the liver by the lesser omentum (omentum minus) and to the dorsal abdominal wall and rumen by a short greater omentum (omentum majus).

## Background

Laterality defects are very rare in domestic animals. Situs inversus is the most common laterality defect in dogs [1-4] and horses [5]. Primary ciliary dyskinesia (PCD) also known as Kartagener’s syndrome [3,5,6], is thought to be the primary cause of this condition, which is often associated with recurrent chronic respiratory problems including bronchiectasia and chronic rhinitis. The few reports on laterality defects in ruminants deal with post-mortem findings in cattle and sheep [7-11]. There have been only two cases of heterotaxy (“heteros” = other and “taxis” = arrangement) described in cattle and they were referred to as situs inversus [7,8], although at least one of these reports was restricted to intra-thoracic organs, and both were not well documented. The anatomical findings in most of the documented cases [9-11] can be best characterised as situs ambiguus, which can be divided into asplenia (right isomerism) and polysplenia (left isomerism). These conditions are also assumed to be morphological correlates of PCD in humans [12-15]. It has been shown that several gene defects are correlated with PCD and thus with laterality defects [12,16-19]. In humans, PCD is a genetically heterogeneous disorder with an autosomal recessive mode of inheritance in most cases [14,15,19].

To the authors’ knowledge, this is the first detailed case report of a situs ambiguus in a cow. Because not all organs were reversed from their normal positions, and some organs had specific deviations from normal in addition to heterotaxy, this case was not diagnosed as situs inversus. The findings serve to expand our knowledge about the clinical signs, functional implications and topography of internal organs in cattle with situs ambiguus.

## Case presentation

### Animal and methods

A three-year-old, non-pregnant primiparous Brown Swiss cow, which had previously produced a live calf was referred to the Department of Farm Animals, Vetsuisse Faculty, University of Zurich, because of poor body condition and a tentative diagnosis of caecal dilatation. The cow was small for its age and therefore bred late, resulting in an age at first calving of 39 months. The cow was often seen ruminating but had a history of poor appetite, and production during early lactation was two thirds of the herd average. The cow resumed ovarian cyclicity and was inseminated twice post partum but did not conceive. The animal underwent clinical and ultrasonographic examinations and blood was collected for haematological and biochemical analyses. Haematocrit, total leukocyte count and the concentrations of fibrinogen and total protein from EDTA blood samples were determined on an automated blood analyser (CELL-Dyn 3500, Abbott Diagnostics Division, Baar). The concentrations of serum bilirubin, urea nitrogen, sodium, chloride, potassium, calcium, inorganic phosphorus and magnesium were determined at 37°C using an automated analyser (Cobas-Integra-800-Analyser, Roche Diagnostics, Basel) and the manufacturer’s reagents (Roche-Reagents) according to the International Federation of Clinical Chemistry and Laboratory Medicine (IFCC). Rumen fluid was collected using a stomach tube and the chloride concentration measured with an MK-II-Chloride Analyser 9265 (Sherwood, Cambridge). Results were compared with reference values established at this clinic.

A standing right-flank exploratory laparotomy was done because of apparent abnormalities in the topography of the abdominal organs, suspected caecal dilatation and left displacement of the abomasum. During laparotomy it was confirmed that the abdominal organs were arranged in a mirror image reversal of the normal positioning. The left dorsal displacement of the abomasum was corrected and the general condition of the patient improved and the cow was discharged five days postoperatively.

The cow was re-admitted to the clinic 2.5 months later because she failed to gain weight and milk production remained poor. Because of a grave prognosis, the cow was euthanised, exsanguinated, fixed in standing position with 2.8% formaldehyde administered through the common carotid artery (arteria carotis communis), and necropsied. Samples of the mucosa of nasal conchae (conchae nasales), frontal sinus (sinus frontalis) and trachea were collected and routinely processed for scanning and transmission electron microscopy for the examination of the presence and structural integrity of ciliated cells. Samples of liver tissue and of two control livers of non-liver-diseased cows ageing five and six years were collected, fixed in 10% formalin, embedded in paraffin and routinely processed for light microscopy and immunohistochemistry. Staining with haematoxylin and eosin, Gomori’s blue trichrome (Artisan™, Dako) and reticulin-nuclear fast red stains (Foot, Artisan™, Dako), histochemical detection of bilirubin (according to Hall), copper (rhodamine method for copper) and ferric iron pigments (Iron stain, Artisan™, Dako) and immunohistochemical detection of α-smooth muscle actin (monoclonal mouse anti-human alpha muscle actin, clone 1A4, Dako) and desmin (monoclonal mouse anti-human desmin, clone D33, Dako) were carried out at the Institute of Veterinary Pathology, University of Zurich using standard protocols. Cytokeratin (mouse anti-human cytokeratin, MNF1166, Dako) and von Willebrand factor immunohistochemistry (polyclonal rabbit anti-human von Willebrand factor, Dako) were done to distinguish vessels from bile ducts. The size of 20 liver lobules from this cow as well as from two healthy controls, aged five and six years, was measured histomorphometrically using AxioVison software (Release 4.63, Zeiss), means and standard deviations were calculated, and differences analysed using an unpaired *t*-test (Statview 5.0 for Windows).

### Clinical findings during the first hospitalisation

Auscultation on the left revealed no ruminal contractions and showed that the content of the digestive tract exhibited no layered arrangement at this site. Faeces had normal consistency and colour.

Ultrasonographic examination revealed that the heart had a normal size and shape and the large vessels were positioned normally within the thoracic cavity (cavum thoracis). The rumen was large and gas-filled and only a few loops of the small intestine (intestinum tenue) could be seen on the right side of the abdominal cavity. There were three reticular contractions during a two-minute period. The liver was visible ventrally on the left side of the abdominal cavity and the gall bladder (vesica fellea) was moderately enlarged.

Exploratory laparotomy revealed a heterotaxy of abdominal organs. The rumen was almost empty and situated on the right side of the abdominal cavity. The reticulum was attached to the rumen cranioventrally and contained sand. The omasum was to the left of the rumen and had a firm consistency. The abomasum had a normal size, was on the left side and displaced dorsally. It contained a small amount of firm and doughy ingesta. The intestines were to the left of the rumen, almost empty and had good peristalsis. The distal half and the tip of the caecum (apex caeci) had a normal size and shape. The greater omentum, which normally forms the supraomental recess (recessus supraomentalis) containing the intestines in ruminants, was not detected during surgery (see post-mortem findings). The abomasum was repositioned ventrally in the abdomen before closure of the abdominal wall.

Serum biochemistry analysis on the day of initial hospitalisation showed a slightly increased bilirubin concentration as well as increased activities of glutamate dehydrogenase, aspartate aminotransferase and creatine kinase. The activity of sorbitol dehydrogenase was moderately increased and the activity of gamma-glutamyltransferase was severely elevated. The cow also had a hypokalaemic and hypochloraemic metabolic alkalosis. The chloride concentration of the ruminal fluid was normal (Table 1). 

**Table 1:** Biochemical analysis of selected variables in serum and ruminal fluid

**Variable**	**Result**	**Reference range**
Total bilirubin	3.9 μmol/l	1.5-2.9 μmol/l
Glutamate dehydrogenase	22.7 U/l	4.0-18.2 U/l
Aspartate aminotransferase	149 U/l	57-103 U/l
Creatine kinase	396 U/l	70-169 U/l
Sorbitol dehydrogenase	16.6 U/l	4.0-7.4 U/l
γ-glutamyltransferase	89 U/l	13-32 U/l
Potassium	3.8 mmol/l	3.9-5.0 mmol/l
Chloride (serum)	98 mmol/l	100-108 mmol/l
Chloride (ruminal fluid)	21 mmol/l	<25 mmol/l

The general condition of the cow improved postoperatively, and one to two ruminal contractions per minute could be ausculatated one day after surgery. The cow was discharged five days postoperatively, but was readmitted 2.5 months later because of failure to gain weight and poor milk production.

### Clinical findings during the second hospitalisation

The findings after re-admission to the clinic were similar to those of the initial physical examination with regard to the forestomach, abomasum, which still had a ventral position, and intestines. Percussion and ultrasonography revealed that the liver was located in the ventral median part of the abdominal cavity directly caudal to the diaphragm.

### Post-mortem findings

#### Respiratory system

**Figure 1:**
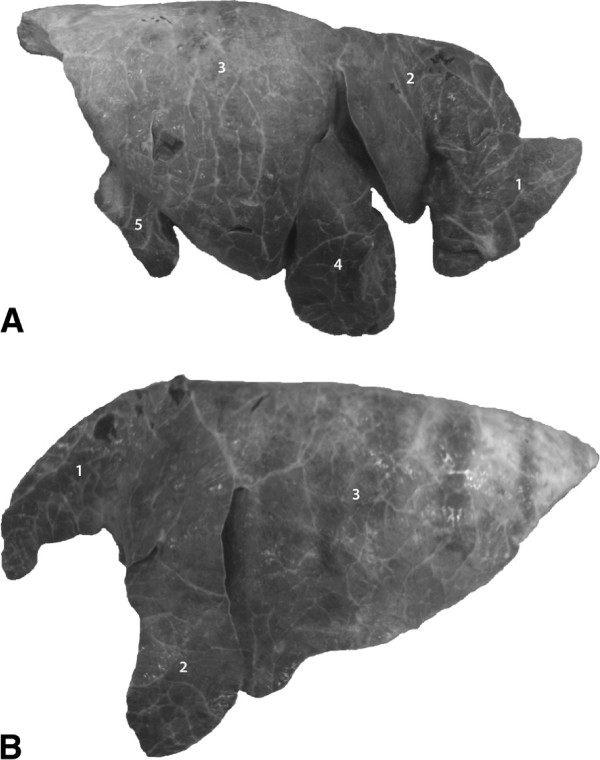
**Right (A) and left (B) lungs, lateral view.** 1 Cranial lobe, cranial part; 2 Cranial lobe, caudal part; 3 Caudal lobe; 4 Middle lobe; 5 Accessory lobe, reduced size. Note the apex of the cranial part of the cranial lobe in A, which was bent dorsally resulting in a V-shaped appearance.

There were no macroscopic signs of acute or chronic inflammation of the mucosa of the nasal cavity, frontal sinus, larynx and trachea. Both lungs were of normal size and had only two anomalies (Figure 1): the apex of the cranial part of the cranial lobe of the right lung (pars cranialis lobus cranialis pulmonis dextri) was bent dorsally resulting in a V-shaped appearance. The accessory lobe (lobus accessorius pulmonis dextri) was small. Light and transmission electron microscopy revealed normal tracheal and nasal ciliary morphology and ultrastructure. The number of cilia-bearing cells varied among different specimens and within single specimens. Scanning electron microscopy showed a few epithelial cells with long and thick apical protrusions representing clotted cilia. Adjacent to these cells were epithelial cells that had intact cilia and some that had microvilli. 

#### Digestive system

**Figure 2:**
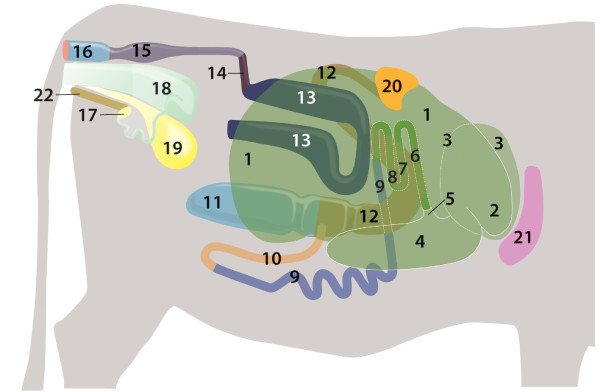
**Relationship of abdominal organs, drawing, right view.** 1 Rumen; 2 Reticulum; 3 Omasum, located left of reticulum; 4–5 Abomasum: 4 Body, 5 Pylorus; 6–8 Duodenum (left side): 6 Initial straight part, 7, 8 U-shaped loop; 9 Jejunum (left side); 10 Ileum (left side); 11 Caecum (left side); 12–13 Ascending colon (left side): 12 Proximal loop, 13 Distal loop; 14 Transverse colon; 15 Descending colon; 16 Rectum; 17 Right ovary; 18 Uterus; 19 Urinary bladder; 20 Right spleen; 21 Liver; 22 Pelvic symphysis.

**Figure 3:**
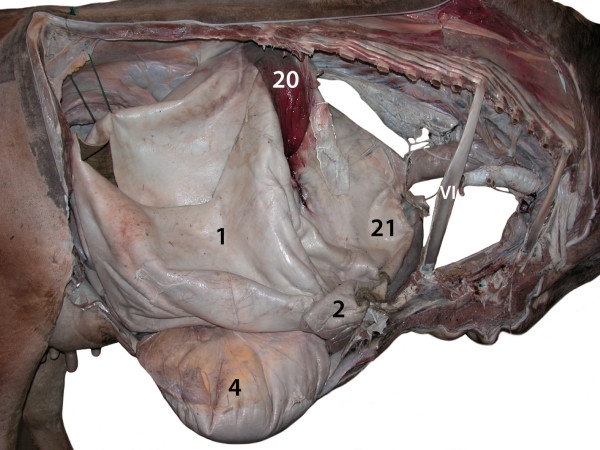
**Abdominal and thoracic cavities, lungs, diaphragm and heart removed, right view.** 1 Rumen; 2 Reticulum; 4 Abomasum; 20 Right spleen; 21 Liver; VI 6^th^ rib.

**Figure 4:**
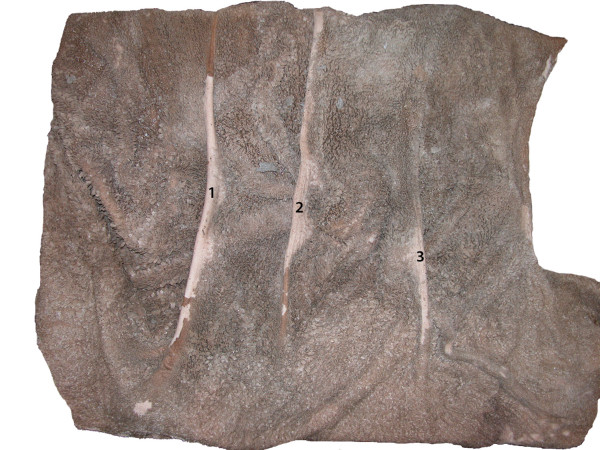
**Rumen interior, dorsal view, cranial is right.** Normal compartmentalization by pillars is lacking. Note three transverse folds 1–3 on floor of rumen.

The anatomy and topography of the digestive tract from the oral cavity to the diaphragm was normal. Within the abdominal cavity there were striking deviations relating to the shape and position of the digestive organs. The reticulum and rumen occupied the right abdomen (Figures 2 and 3). The reticulum was cranial and to the left of the rumen. The reticular groove (sulcus reticuli) was 31 cm long. The reticular mucosa was normal with characteristic polygonal cells. The rumen had a normal mucosa but lacked pillars (pila ruminis) and hence lacked compartmentalisation. However three low transverse folds were noted on the interior of the floor (Figure 4). These folds, from cranial to caudal, were 39, 64 and 69 cm long and the distances between them were 36 and 16 cm respectively. Cranially, the rumen was attached to the dorsal wall of the abdominal cavity.

**Figure 5:**
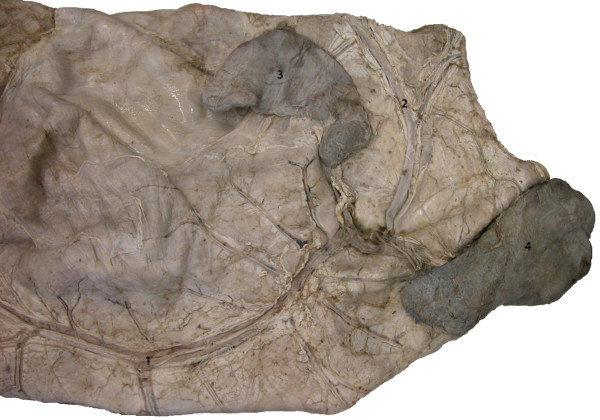
**Rumen with two spleens, dorsal view, cranial is right.** 1 Right ruminal artery and vein; 2 Left ruminal artery; 3 Left spleen; 4 Right spleen.

**Figure 6:**
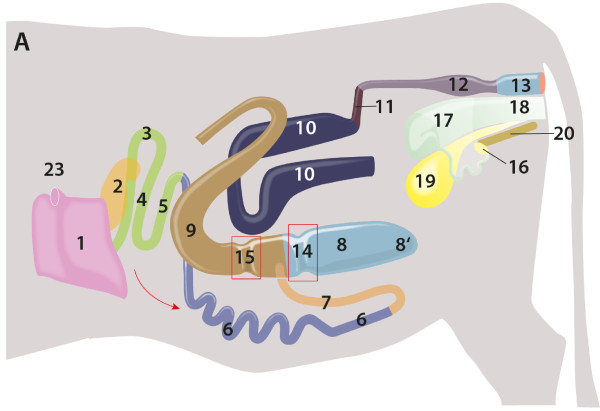
**Relationship of abdominal organs, drawing, stomach and spiral colon removed, all organs located at the left side, left view.** 1 Liver, Left lobe; 2 Left spleen; 3–5 Duodenum: 3 Initial straight part, 4, 5 U-shaped loop; 6 Jejunum; 7 Ileum; 8–8′: Caecum, 8 Body, 8′ Apex; 9–10 Ascending colon: 9 Proximal loop, 10 Distal loop; 11 Transverse colon; 12 Descending colon; 13 Rectum; 14–15 Strictures: 14 in Caecum, 15 in Proximal loop; 16 Left ovary; 17 Uterus; 18 Vagina; 19 Urinary bladder; 20 Pelvic symphysis; 23 Caudal vena cava.

**Figure 7:**
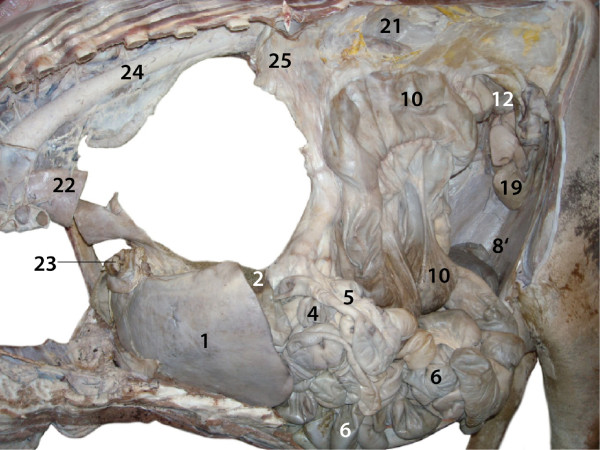
**Abdominal and thoracic cavities, lungs, diaphragm and heart removed, left view.** 1 Liver, Left lobe; 2 Left spleen; 4–5 Duodenum, U-shaped loop; 6 Jejunum; 8′ Apex of the Caecum; 10 Ascending colon, Distal loop; 12 Descending colon; 19 Urinary bladder; 21 Left kidney; 22 End of Esophagus; 23 Caudal vena cava, arising from Left and Right hepatic veins; 24 Descending aorta; 25 Mesenteric root.

Two spleens lay on and were attached to the rumen craniodorsally and to the dorsal abdominal wall. The larger spleen measured 26.5 × 12.0 cm and was on the left and the small right one measured 20.5 × 5.5 cm (Figure 5). The cranioventral wall of the rumen was connected to the abomasum by a short peritoneal fold of the greater omentum. The omasum was cranioventrally and to the left of the rumen and caudal to the liver (Figure 2). The abomasum was on the abdominal floor in a bent position ventral to the omasum and the rumen (Figures 2 and 3). It was loosely attached to the liver by mesogastrium, which originated from the lesser curvature (curvatura minor) of the abomasum and represented the lesser omentum (omentum minus). The abomasal greater curvature (curvatura major) was orientated caudally (Figures 2 and 3) and was connected loosely to the cranioventral aspect of the rumen and the dorsal abdominal wall by the greater omentum (omentum majus). Thus the ventral part of the rumen was not enclosed by the greater omentum, which normally forms the omental bursa (bursa omentalis) and is attached to the abomasum (Figures 3, 6 and 7).

**Figure 8:**
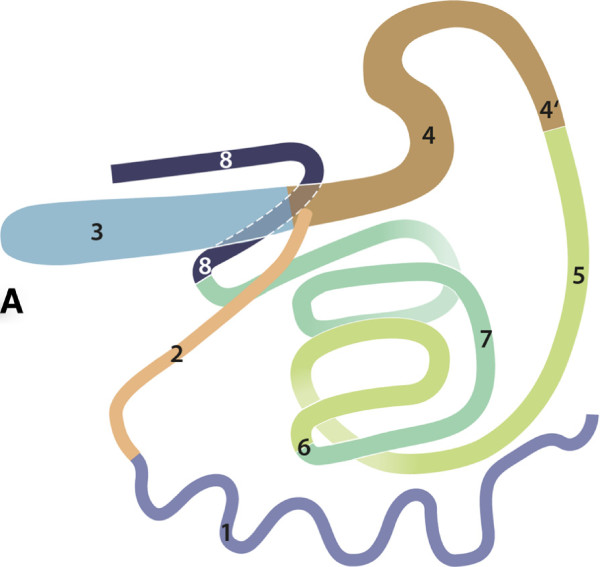
**Relationship of abdominal organs, drawing, stomach and duodenum removed, all organs located on left side, right view.** 1 Jejunum; 2 Ileum; 3 Caecum; 4–8 Ascending colon: 4 Proximal loop, 4′ End of Proximal loop; 5–7 Spiral loop: 5 In-going coils, 6 Central flexure, 7 Out-going coils; 8 Distal loop (left side).

The duodenum had an initial straight part arising from the pylorus and running dorsally. It was continued by a U-shaped loop extending to the left cranial side of the abdominal cavity (Figures 6 and 7). The jejunum was in the left ventral quadrant of the abdominal cavity and formed many short coils. The mesojejunum was not fused with the mesocolon ascendens (Figures 6 and 7). The ileum arose from the jejunum in the left caudoventral abdominal cavity and ran cranially to join the large intestine at the ileal opening (ostium ileale) just cranial to the caecocolic opening (ostium caecocolicum) (Figures 2, 6 and 8). 

**Figure 9:**
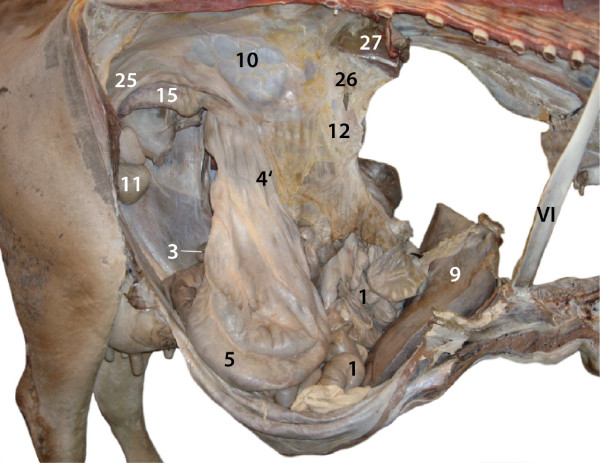
**Abdominal and thoracic cavities, stomach, lungs, diaphragm and heart removed, right view.** 1 Jejunum; 3 Caecum; 4–8 ascending colon: 4′ end of Proximal loop; 5 In-going coils of Spiral loop; 9 Liver, Right lobe; 10 Right kidney; 11 Urinary bladder; 12 Cranial mesenteric artery; 25 Broad ligament; 26 Mesenteric root; 27 Diaphragm, Right crus; VI 6^th^ rib.

The caecum was located on the left side near the median plane at mid-abdominal level with its tip extending caudally (Figures 6, 7, 8 and 9). The ascending colon could be divided into proximal, spiral and distal loops (ansa proximalis, spiralis, and distalis coli) (Figures 7, 8 and 9). The proximal loop was S-shaped and continued cranially on the right as spiral loop, which was situated near the median plane. The mesojejunum was not fused with the part of the mesocolon ascendens that fixes the spiral loop. The normally flattened, discoid and sagittally extending spiral loop was folded such that the cranial and caudal rims of the disc were fused, which resulted in a characteristic bi-layered intestinal complex (Figures 8 and 9). The caudal margin of the spiral loop was thus adjacent and fixed to its cranial margin resulting in a semicircular structure. The distal loop was U-shaped, started with a cranially running section, had an ascending cranial loop and led to the transverse colon (colon transversum) dorsocaudally (Figures 7 and 8). There were two fibrosed strictures associated with the large intestine. One involved the caecum and the other involved the beginning of the proximal loop of the ascendic colon (Figure 6). The transverse colon crossed the midline from the left to right caudal to the cranial mesenteric artery (arteria mesenterica cranialis). The descending colon (colon descendens) originated on the right side, but the anatomy and position of the remainder of the descending colon as well as the rectum were normal.

The mesenteric root (radix mesenterii) was situated in the midline between the crura of the diaphragm and cranial to the kidneys (Figures 7 and 9).

**Figure 10:**
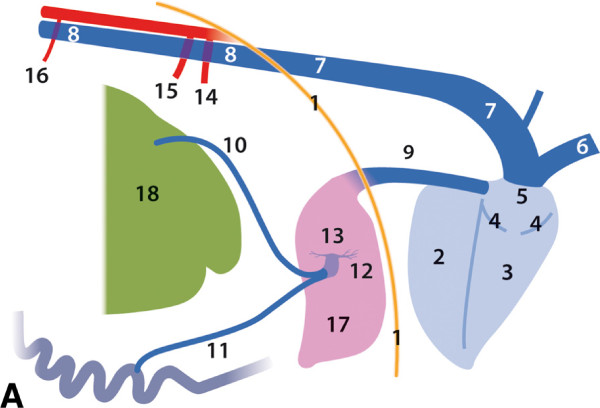
**Relationship of selected abdominal and thoracic organs, drawing, right view.** 1 Diaphragm; 2–5 Heart: 2 Left ventricle, 3 Right ventricle; 4 Right atrioventricular (tricuspid) valve; 5 Right atrium; 6 Cranial vena cava; 7 Right azygous vein, enlarged; 8 Caudal vena cava, draining into the Right azygous vein; 9 Caudal vena cava; 10, 11 Veins draining the gastrointestinal organs; 12 Portal vein; 13 Hepatic branches; 14 Coeliac artery; 15 Cranial mesenteric artery; 16 Caudal mesenteric artery; 17 Liver; 18 Rumen.

**Figure 11:**
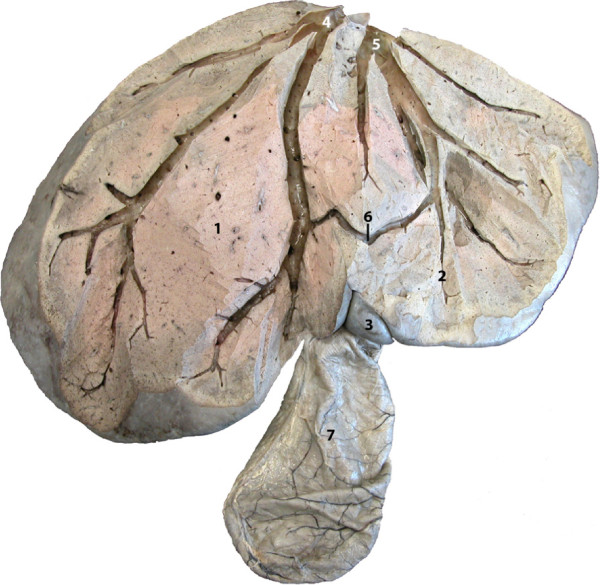
**Liver, cranial view, after dissection of the venous drainage.** 1 Right lobe; 2 Left lobe; 3 Quadrate lobe; 4 Right hepatic vein; 5 Left hepatic vein; 6 Anastomosis between 4 and 5; 7 Gall bladder.

The liver had transversally orientated dorsal (margo dorsalis) and ventral borders (margo ventralis), a ventromedian position immediately caudal to the diaphragm (Figures 6, 7, 9 and 10) and was composed of two large lobes and a very small lobe (Figure 11, see Additional file 1: Figure S1 and Additional file 2: Figure S2). The right lobe (lobus dexter) was larger than the left lobe (lobus sinister). The very small lobe was located infraportally between the larger two lobes, cranial and right to the round ligament (ligamentum teres hepatis), right to the fissure of the round ligament (fissura ligamentum teretis) and the falciform ligament (ligamentum falciforme hepatis) and left and cranial to the gallbladder (vesica fellea) and the cystic duct (ductus cysticus). This small and cylindric lobe had a triangular basis on the visceral side, extended to the ventral border (margo ventralis) and the diaphragmatic side of the liver demonstrating a triangular rounded and dome-like shape (Figure 11, see Additional file 2: Figure S2). The dorsal (margo dorsalis) and the ventral borders (margo ventralis) of the liver were transversally orientated.

**Figure 12:**
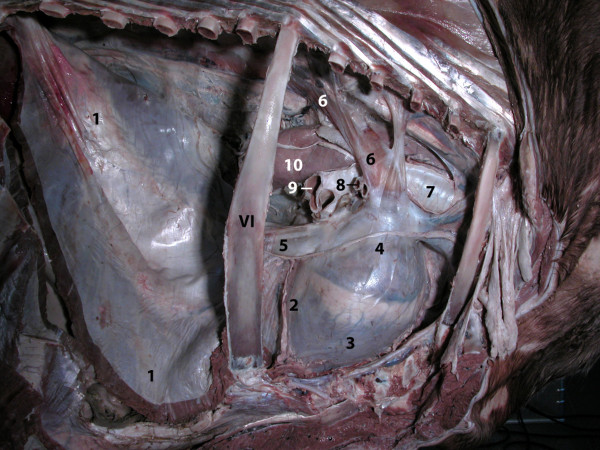
**Thoracic cavity, lung removed, right view.** 1 Diaphragm; 2–4 Heart: 2 Left ventricle, 3 Right ventricle; 4 Right atrium; 5 Caudal vena cava; 6 Right azygous vein, enlarged; 7 Trachea; 8 Tracheal bronchus; 9 Division of the Chief bronchus into Caudal and Middle bronchus; 10 Oesophagus; VI 6^th^ rib.

The liver received blood from two large gastrointestinal veins (Figure 10, see Additional file 3: Figure S3), which joined to form a very short (7.0 cm) portal vein (vena portae) with a large diameter (3.5 cm), which was subcapsular and nearly completely intrahepatic (Figure 10, see Additional file 3: Figure S3 and Additional file 4: Figure S4). Two large branches were dispatched (right 15 mm, left 7 mm) (Figure 10, see Additional file 4: Figure S4) which supplied their respective lobes. Many small branches (2 to 4 mm in diameter) arose from the two large hepatic branches, as well as, from the portal vein (Figure 10, see Additional file 4: Figure S4). The round ligament, the falciform ligament, the gallbladder and the cystic duct were in close proximity. The gallbladder was slightly enlarged (Figure 11, see Additional file 1: Figure S1). Left and right hepatic veins (vena hepatica sinstra and dextra), joined to form the caudal vena cava. A large anastomosis was present near the ventral border of the liver (Figure 11). The caudal vena cava - arising solely from the two major hepatic veins in this animal - passed through the diaphragm via the caval foramen (foramen venae cavae) and joined the right atrium of the heart (Figures 10 and 12). 

**Figure 13:**
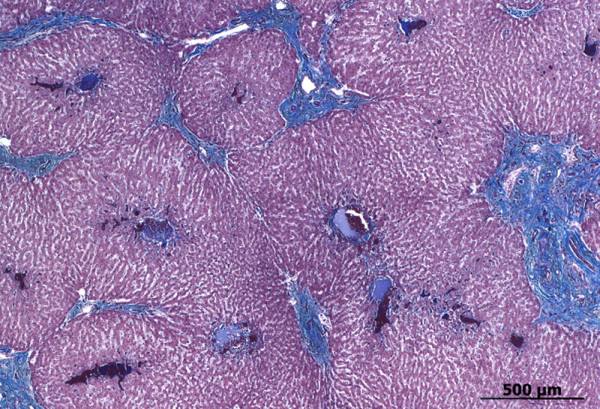
**Photomicrograph of liver.** Gomori’s blue trichrome stain demonstrates increased periportal connective tissue.

Histologically, the hepatic lobules (lobuli hepatici) had a normal architecture (Figure 13, see Additional file 5: Figure S5), but had two or three central veins (venae centrales) compared with only one in control livers. The width of the hepatic lobules (radius = 461 ± 150 μm) was significantly (p < 0.0001) reduced compared with two control livers (672 ± 88 μm and 710 ± 184 μm). The enlarged portal fields (see Additional file 6: Figure S6) had two to six arterioles, one thin-walled venule and one or several lymphatic vessels, which were sometimes mildly dilated. Moderate periportal fibrosis, consisting of coarse collagen fibres and proliferating fibroblasts (Figure 13, see Additional file 6: Figure S6), and moderate hyperplasia of small biliary ductules (ductuli biliferi) were also prominent features (see Additional file 6: Figure S6). There were relatively few inflammatory cells (mainly lymphocytes and plasma cells), which was similar to the control livers. The number of biliary ductules was increased but the ductual lumina were normal (see Additional file 6: Figure S6). There was no increase in connective tissue or reticular fibres intralobularly (Figure 13, see Additional files 7: Figure S7 and 8: Figure S8). However, a thick mesh of actin filaments was diffusely present along the sinusoids (see Additional file 9: Figure S9), but not in the controls (see Additional file 10: Figure S10). There was no increased immunoreaction against desmin. No bilirubin, copper and ferric iron pigments were detected in the adjacent hepatocytes. 

#### Cardiovascular system

The anatomical features and position of the heart were normal. The abdominal cavity demonstrated one striking abnormality of the venous drainage of the trunk, pelvic limbs, kidneys, and pelvic organs. This large venous trunk (caudal vena cava) did not descend to the dorsal margin of the liver, drain the liver, pass through the diaphragm and end in the right atrium of the heart. Instead it connected with the right azygous vein as it passed between the crura of the diaphragm, below the vertebral column (columna vertebralis) on the right surface of the descending aorta (aorta descendens). This enlarged right azygous vein drained into the right atrium of the heart (Figures 10 and 12, see Additional file 11: Figure S11).

Both ruminal arteries (Aa. ruminales dextrae et sinistrae) run along the craniodorsal wall of the rumen to the right and left side of this organ and proceeded caudally giving rise to dorsal and ventral branches (Figure 5).

#### Urogenital system

Both kidneys were positioned retroperitoneally; the left kidney was further cranial than the right kidney, which represented an abnormal position (Figures 7 and 9).

## Discussion

In ruminants, the most common manifestation of heterotaxy of internal organs appears to be situs ambiguus, which is divided into two primary subtypes: Asplenia syndrome or right isomerism, and Polysplenia syndrome or left isomerism [9-11]. Situs inversus, however, describes a situation in which all visceral organs are reversed or mirrored from their normal position (referred to as situs solitus [13]) and is considered extremely rare in ruminants [7,8]. To our knowledge, there have been three cases of asplenia, six cases of hypoplastic spleens, 15 cases of two spleens and only two cases of situs inversus described in cattle [7,8,10,11]. The main findings in our patient (continuation of the caudal vena cava into the right azygos vein, polysplenia, continuation of a common hepatic vein into the right atrium, heterotaxia of the digestive tract, tri-lobed liver, retroperitoneal position of both kidneys, positioning of the left kidney cranial to the right kidney and normal anatomy and topography of heart and lungs) were in general agreement with other reports of polysplenia syndrome in cattle [10,11] und thus strongly indicate the presence of this syndrome in our cow. Characteristics of the polysplenia syndrome in cattle - as reported in literature and detected in this animal - are: continuation of the vena cava caudalis into the vena azygos dextra (n = 6; number of cases) or sinistra (n = 3), continuation of a common hepatic vein into the atrium dextrum (n = 4), situs inversus (n = 2) and left isomerism (n = 4) of the liver, situs inversus of the stomach (n = 2), retroperitoneal position of both kidneys (n = 1), positioning of the left kidney cranial to the right kidney (n = 1) and normal anatomy and topography of the lungs [10,11].

Primary ciliary dyskinesia has been associated with chronic airway diseases and impaired fertility in dogs, horses, and humans [1-3,5,13-15,20]. It is an unlikely cause of heterotaxy in cattle because in the patient described in this report, there was no chronic respiratory disease and the ultrastructure of the cilia was normal. Although fertility was severely reduced in this cow, this does not support an etiological role of PCD since the cow was inseminated late because of small size and did therefore produce a calf at 39 months of age. Because genetic and functional analyses were not carried out, PCD cannot be ruled out in this case.

In humans, neonates with polysplenia syndrome have a high incidence of severe heart disease and therefore high juvenile mortality. Only children without severe cardiovascular defects and those in which cardiovascular defects are corrected immediately after birth survive and reach adulthood. Therefore, in adults, polysplenia syndrome is clinically characterised by gastrointestinal heterotaxia and non-life-threatening vascular abnormalities [13,15,18,21,22], which is similar to findings in our patient and in accordance with reports in the literature [10,11]. Further investigations in neonate calves are needed to determine whether similar cardiovascular defects occur in cattle with polysplenia or asplenia syndrome; unfortunately most calves that die shortly after birth because of postnatal asphyxia or cyanosis are not necropsied.

Many of the topographical abnormalities observed in the present case can be explained by specific developmental stages of organs during embryogenesis. The topography of the liver in our patient was characteristic for the position of this organ in the perinatal period in cattle, indicating that the ruminant-specific shift to the right side and anti-clockwise quarter-turn of the organ during the postnatal life had not occurred [23]. The caudal vena cava is usually composed of a sequence of several sagittal embryonic vessels [24]. The lack of connection or a faulty connection between the intra-hepatic or immediate post-hepatic parts of these sagittal veins results in a dorsal anastomosis between the right or left azygous vein and the abdominal segment of the caudal vena cava. The latter vein therefore does not descend to the dorsal margin of the liver. This modification of vasculogenesis leads to a continuation of the caudal vena cava into the right azygous vein, which occurred in our patient and also in several other cattle [11]. The only function of the final segment of the caudal vena cava, which ran from the liver and passed through the diaphragm to join the right atrium of the heart, was drainage of the liver. From a functional point of view, it served as a common hepatic vein.

The asymmetrical morphology of the tri-lobed liver was reflected by the specific drainage of the two large lobes by two large hepatic veins, one on the right and one on the left. This finding was also in agreement with the very small size of a liver lobe that possibly represent the quadrate lobe and the complete absence of the caudate lobe (lobus caudatus), which are normally drained by the large middle hepatic vein (vena hepatica media) [25]. The specific anatomy of the liver and its midline position in the abdominal cavity support the concept of a polysplenia syndrome/left isomerism in this animal.

The histological characteristics of the hepatic lobules were indicative of abnormal haemodynamics. However, different types of haemodynamic changes may cause identical stereotypical abnormalities in the portal triad (pattern of portal vein hypoperfusion) similar to those described in our patient. Causes of portal vein hypoperfusion include congenital portosystemic shunts, arterioportal fistulas and obstruction or hypoplasia of the portal vein. The macroscopic and histological changes in the vasculature of our patient represented primary pre- and intra-hepatic hypoplasia of the portal vein [26]. The changes in liver microstructure may also have reflected or caused the poor nutritional status of this cow. Normal liver function is a prerequisite for many catabolic and anabolic processes, and abnormalities in serum enzyme activities and bilirubin concentration in this cow were characteristic of chronic impairment of liver function [27]. There have been no previous reports of the histological changes in the liver of ruminants with situs ambiguus.

## Conclusions

In the present case report, there were striking abnormalities in the anatomy, topography, fixation and thus mobility of the abdominal parts of the digestive tract, which resulted in obvious digestive malfunction, small body seize, poor body condition and low milk production of the cow.

These findings seen in our patient may have been caused by a number of factors including the loose connection of the abomasum to the cranially situated abdominal organs and wall and thus rendering susceptibility to dislocation and bloating, constrictions of the large intestine at the level of the caecum and the proximal loop of the ascending colon, and lack of normal ruminal compartmentalisation. The laboratory findings suggested reduced feed intake caused by mild abomasal reflux syndrome and/or prolonged periods of recumbency. Whether liver abnormalities contributed to poor body condition is not clear. However, marked fibrosis of the liver reflected poor nutritional status and possibly chronically impaired metabolism. The absence of inflammation and cholestasis and the presence of arteriolar proliferation, hypoplastic venules, portal fibrosis and bile duct proliferation were indicative of abnormal haemodynamics.

It was surprising that the cow was able to produce a live calf, given that fertility was obviously impaired. Equally surprising was that the cow had ruminal contractions and was seen by the owner to ruminate given the ruminal anomalies observed. The normal consistency of the faeces was an indication of a certain level of normalcy of digestive tract function.

### Consent

Consent was obtained from the owner of the cow for publication of this case report and any accompanying images.

## Competing interests

The authors declare that they have no competing interests.

## Authors’ contributions

AB carried out and supervised the postmortem examination of the cow’s thorax and stomach, documented the postmortem findings, drafted major parts of the manuscript and searched and reviewed the literature. HG carried out and supervised the postmortem examination of the cow’s intestines, designed the schematic illustrations and drafted parts of the manuscript (the section on the intestines). UM carried out the postmortem examination. JP designed and made the schematic illustrations. TaS and MP carried out the clinical examinations and surgery. MR and TiS did the histological and immunohistochemical evaluations and drafted the corresponding parts of the manuscript. CCS and CG were responsible for ultrasonography of the heart and abdomen, respectively. EMS carried out and validated the ultrastructural assessments. UB initiated and supervised the case report, performed the clinical examination, initiated surgery and supported preparation of the manuscript. All authors read and approved the final manuscript.

## Supplementary Material

Additional file 1: Figure S1 Liver, caudal view. 1 Left lobe; 2 Right lobe; 3 Minor omentum; 4 Caudal vena cava with adherent rim of diaphragmatic tissue; 5 Gall bladder. Click here for file

Additional file 2: Figure S2 Liver, cranial view. 1 Right lobe; 2 Left lobe; 3 Quadrate lobe; 4 Caudal vena cava with adherent rim of diaphragmatic tissue; 5 Falciform ligament. Click here for file

Additional file 3: Figure S3 Liver, hilus, caudal view. 1 Hepatic lymph node; 2 Hepatic artery; 3 Bile duct; 4, 4’ veins draining gastrointestinal organs. Click here for file

Additional file 4: Figure S4 Liver, hilus dissected. 1 Liver, Left lobe; 2 Liver, Right lobe; 3 Portal vein; 4 Left branch and 5 Right branch of 3; 6 Right branch of the hepatic artery; 7 Common hepatic duct; 8Cystic duct; 9 Gall bladder; 10 End of gastrointestinal veins. Click here for file

Additional file 5: Figure S5 Photomicrograph of liver. Haematoxylin and eosin stain demonstrates hepatic lobules, duplications of central veins (arrows) and periportal fibrosis (arrowheads). Click here for file

Additional file 6: Figure S6 Photomicrograph of liver. Haematoxylin and eosin stain demonstrates a portal tract with fibrosis, proliferation of bile ducts (star), increased numbers of small arteries (arrow), and several cross sections of small thin-walled hepatic veins or lymphatic vessels (arrowhead). Click here for file

Additional file 7: Figure S7 Photomicrograph of liver. Reticulin-nuclear fast red stain (Foot) demonstrates large amount of reticulin fibres within the portal tract. Click here for file

Additional file 8: Figure S8 Photomicrograph of control liver. Reticulin stain (Foot) demonstrates a small amount of reticulin fibres along the sinusoids. Click here for file

Additional file 9: Figure S9 Photomicrograph of liver. Immunohistochemical staining of alpha smooth muscle actin demonstrates large amounts of actin filaments in the portal tracts, interlobularly and along the sinusoids. Click here for file

Additional file 10: Figure S10 Photomicrograph of control liver. Immunohistochemical staining of alpha smooth muscle actin demonstrates a small amount of signal along the sinusoids. Click here for file

Additional file 11: Figure S11 Thoracic cavity, lung removed, right view. 1 Right azygous vein, enlarged; 2 Middle mediastinal lymph node; 3 Caudal mediastinal lymph node; 4 Thoracic duct; 5 Descending aorta; 6 Sympathetic trunk; 7 Dorsal branch of the right vagus nerve; 8 Tracheal bronchus; 9 Right chief bronchus; 10 Oesophagus. Click here for file
